# Alterations of Serum Levels of BDNF-Related miRNAs in Patients with Depression

**DOI:** 10.1371/journal.pone.0063648

**Published:** 2013-05-21

**Authors:** You-Jie Li, Mei Xu, Zong-Hua Gao, Ya-Qi Wang, Zhen Yue, Yan-Xia Zhang, Xin-Xin Li, Can Zhang, Shu-Yang Xie, Ping-Yu Wang

**Affiliations:** 1 Department of Biochemistry and Molecular Biology, Binzhou Medical University, YanTai, ShanDong, P.R. China; 2 Department of Psychiatry of Yantai Fushan District People’s Hospital, YanTai, ShanDong, P.R. China; 3 Department of Chemistry, Binzhou Medical University, YanTai, ShanDong, P.R. China; 4 Genetics and Aging Research Unit, Department of Neurology, Massachusetts General Hospital and Harvard Medical School, Charlestown, Massachusetts, United States of America; 5 Department of Epidemiology, Binzhou Medical University, YanTai, ShanDong, P.R. China; Chiba University Center for Forensic Mental Health, Japan

## Abstract

Depression is a serious and potentially life-threatening mental disorder with unknown etiology. Emerging evidence shows that brain-derived neurotrophic factor (BDNF) and microRNAs (miRNAs) play critical roles in the etiology of depression. Here this study was aimed to identify and characterize the roles of BDNF and its putative regulatory miRNAs in depression. First, we identified that miR-182 may be a putative miRNA that regulates BDNF levels by bioinformatic studies, and characterized the effects of miR-182 on the BDNF levels using cell-based studies, side by side with miR-132 (a known miRNA that regulates BDNF expression). We showed that treatment of miR-132 and miR-182 respectively decreased the BDNF protein levels in a human neuronal cell model, supporting the regulatory roles of miR-132 and miR-182 on the BDNF expression. Furthermore, we explored the roles of miR-132 and miR-182 on the BDNF levels in depression using human subjects by assessing their serum levels. Compared with the healthy controls, patients with depression showed lower serum BDNF levels (via the enzyme-linked immunosorbent assays) and higher serum miR-132 and miR-182 levels (via the real-time PCR). Finally, the Pearson’s (or Spearman’s) correlation coefficient was calculated to study whether there was a relationship among the Self-Rating Depression Scale score, the serum BDNF levels, and serum BDNF-related miRNA levels. Our results revealed that there was a significant negative correlation between the SDS scores and the serum BDNF levels, and a positive correlation between the SDS scores and miR-132 levels. In addition, we found a reverse relationship between the serum BDNF levels and the miR-132/miR-182 levels in depression. Collectively, we provided evidence supporting that miR-182 is a putative BDNF-regulatory miRNA, and suggested that the serum BDNF and its related miRNAs may be utilized as important biomarkers in the diagnosis or as therapeutic targets of depression.

## Introduction

Depression is a serious and potentially life-threatening clinical mental disorder with unknown etiology. In some cases, it shows severe and persistent symptoms, and insidiously impacts on the life quality of the patients [1]. Depression is sometimes associated with impaired recovery from a number of medical conditions, including stroke, hip fracture, and myocardial infarction [2]–[4]. Gender has also been found to associate with depression, with women demonstrating higher levels of depressed mood compared with men [5], [6]. Though the molecular and cellular mechanisms of depression have still not been completely elucidated, it was suggested that nearly 50% of the risk for depression is contributed to genetic factors [7]. Furthermore, emerging evidence shows that changes in gene expression play crucial roles in the pathogenesis of depression [8], [9]. The *bdnf* gene is one of the most essential genes which are involved in the pathophysiology of several mental disorders, including depression. The BDNF protein is a neurotrophin and plays essential roles in neuronal development and plasticity. Decreased BDNF protein has been suggested to affect the pathology of major depressive disorder (MDD) [10], [11]. *Bdnf* has also been suggested as a susceptibility gene for MDD and schizophrenia (SCZ), especially in a subgroup of patients with schizophrenia and a lifetime history of depressive symptoms. Moreover, a polymorphism in the *bdnf* gene is associated with depression-related traits in several independent studies [12]–[15]. Because it is difficult to assess the BDNF levels directly from patients’ brain, increasing effort has focused on measuring the BDNF levels in the blood. The serum BDNF levels were found to be lower in depressed patients compared with control subjects [16]. Interestingly, the serum BDNF levels were increased following the treatment of antidepressant medications [17], suggesting that the serum BDNF levels could be as a surrogate biomarker for determining both the depression status and the efficacy of antidepressant treatment.

Considerable effort has been focused on identifying and characterizing the factors that modulate BDNF levels, one of which that has been recently intensively studied is microRNA (miRNA). miRNAs are endogenous small non-coding RNAs that regulate various gene expression at post-transcriptional levels through targeting mRNAs for cleavage or translational repression [18]. miRNAs are essential for different cellular processes, including metabolism, differentiation and apoptosis in both animals and plants [19]–[21]. Increasing evidence suggests a critical role of miRNAs in the pathogenesis of neuropsychiatric disorders, including depression. For example, two polymorphisms, located in pre-miR-182 and pre-miR-30e, were associated with the increased risk of the major depressive disorder [22], [23]. Another study showed that miR-124a, one of the most abundant miRNAs in the brain [24], may contribute to depression by suppressing the expression of glucocorticoid receptor, the activation of which interferes with the hippocampal neurogenesis [25]. In addition, a study showed that either up-regulation of miR-16 in raphe or down-regulation of miR-16 in locus coeruleus induced behavioral responses using depressive mouse models [26]. In summary, these studies clearly showed that miRNAs are involved in the etiology of depression by regulating their targeting genes.

Considering the important roles of BDNF and miRNAs in the pathogenesis of depression, our current study was aimed at identifying the novel miRNAs that regulate serum BDNF levels, and characterizing the relationship between miRNAs and depression. First, in a neuronal cell-based model, we used miR-132 as a positive control and confirmed that it regulated the expression of BDNF as previous report [27]. We also found that miR-182 was a novel putative miRNA that regulated the BDNF expression through bioinformatic and cell-based functional studies. Next, we measured the serum levels of BDNF, miR-132 and miR-182 in human subjects with depression and controls. Our results demonstrated that serum BDNF levels were decreased in depressed patients compared with control subjects, consistent with previous studies. We also found that the levels of miR-132 and miR-182 were increased in patients compared with those in healthy controls. Our results demonstrate that the serum BDNF and its related miRNAs may be utilized as important biomarkers for the diagnosis or as the therapeutic targets of depression.

## Results

### Clinical Characteristics of Patients and Controls

First, we characterized the demographic and clinical characteristics of all the patients and controls that took part in this study (Table 1). Forty patients with depression and forty controls participated in the study. Notably, the patients in this study with melancholic features showed observable psychomotor retardation and all patients diagnosed with psychotic depression showed mood-congruent delusions, like delusions of guilt, delusions of poverty or nihilistic delusions. None of the patients with depression in the present study met criteria for atypical depression or suffered from hallucinations.

**Table 1:** Demographic and clinical characteristics of the study samples (n = 40 in each sample).

	Healthy controls (n = 40)	Patients (n = 40)	*P*-value
Age in years (mean ± SD)	38.35±7.07	39.55±8.66	0.65
Sex (M/F)	21/19	20/20	0.82
SDS score (mean ± SD)	36.19±6.87	59.56±4.87	4.21E-22
BDNF (ng/ml, mean ± SD)	25.22±5.17	20.05±5.98	7.89E-05
miR-132 (10^3^ copies/ml, median)	0.13	0.58	1.96E-04
miR-182 (10^3^ copies/ml, median)	0.01	0.02	2.58E-03

SD, standard deviation. SDS, Self-Rating Depression Scale. BDNF, brain-derived neurotrophic factor. *P* -values are from the Student's t-test or Wilcoxon rank sum test to compare patient samples with healthy control samples.

### Lower Serum BDNF Levels in Patients with Depression

**Figure 1:**
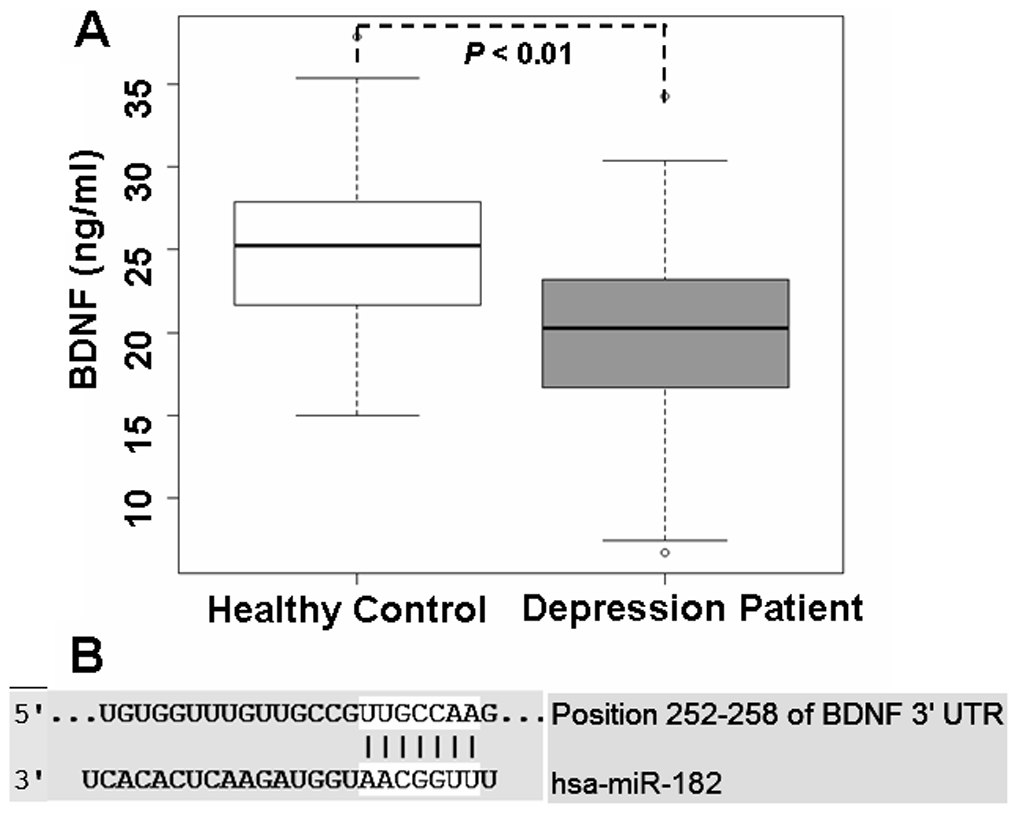
BDNF serum levels and its related miRNAs. (**A**) BDNF concentrations in patients and controls. The serum levels of BDNF in the patients with depression (n = 40; 20.05±5.98 ng/ml) were significantly decreased compared with those of the healthy controls (n = 40; 25.22±5.17 ng/ml). (**B**) The targeting site on BDNF-mRNA-3′UTR by miR-182, which was aligned with the mRNA-3′UTR of human *bdnf* gene with the nucleotide position. Vertical lines indicate identity.

We next studied the serum BDNF levels in patients with depression and controls by ELISA. Our results showed that the mean serum BDNF levels in the patient samples were 20.05±5.98 ng/ml, which were much lower than those in healthy controls 25.22±5.17 ng/ml (Table 1, Fig. 1A). This finding was supported by a previous reported showing that the serum BDNF levels were decreased in patients compared with control subjects [16]. Thus, these independent studies suggest that the serum BDNF levels could be utilized as a surrogate biomarker of depression.

### Higher Levels of BDNF-related miRNAs in Patients with Depression

Next, we explored to identify and characterize putative miRNAs that regulate BDNF protein levels. We utilized an widely-utilized online miRNA analysis software, which provides a comprehensive analysis to identify the targeting genes of specific miRNAs (http://www.microrna.org/microrna/getMirnaForm.do, or http://www.targetscan.org/index.html). We found that the BDNF mRNA 3′-untranslated regions (UTR) was targeted by miR-182 through this computational analysis (Fig. 1B). As a positive control, miR-132 was also investigated in this study, which has been reported to regulate BDNF expression [27].

**Figure 2:**
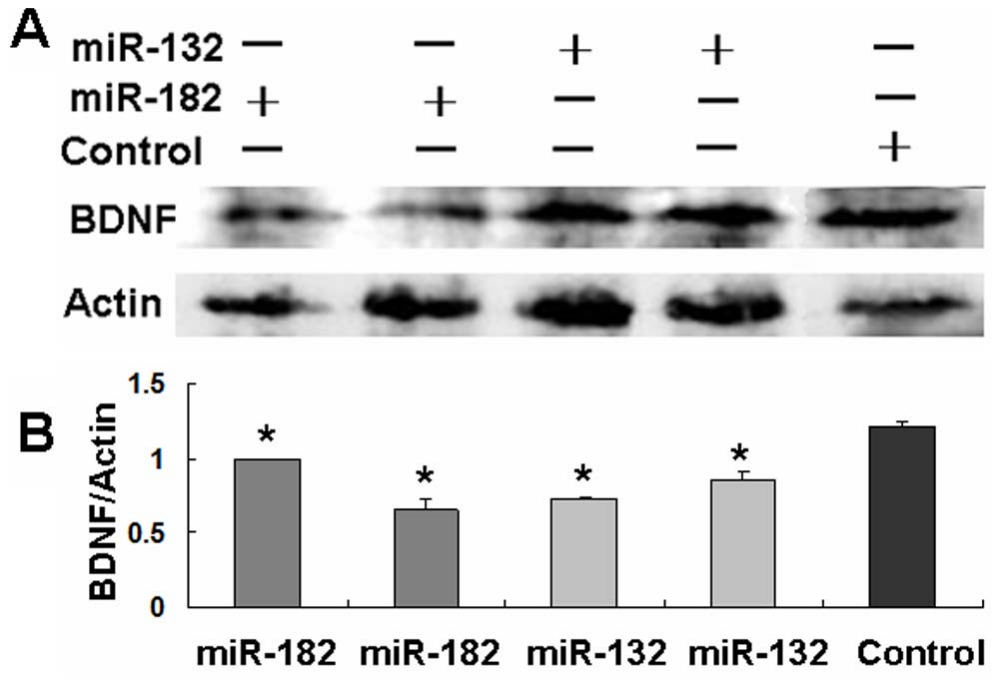
miR-132/182 regulating BDNF expression. (**A, B**) Detection of BDNF by western blotting. SH-SY5Y cells were treated with miR-182 or miR-132, the results showed that BDNF expression in the miR-182- or miR-132-treated cultures was much lower than that of negative control miRNA cultures. *P<0.05, miR-182- or miR-132-treated cultures *vs.* control cultures. Relative values for BDNF *vs*. Actin are indicated in Fig.2B.

In our next set of experiments, we investigated the functional roles of miR-182 in regulating the levels of BDNF using cell-based models. After chemical synthesis of miRNAs, the human neuroblastoma SH-SY5Y cells were transfected with miR-132 or miR-182, and the BDNF expression was detected by western blotting analysis. Our results showed that the BDNF levels were decreased in miR-182-treated cells, as well as in miR-132-treated cultures, compared with control treatment (Fig. 2A,B). Thus, our results supported the roles of both miR-182 and miR-132 in regulating the BDNF expression.

**Figure 3:**
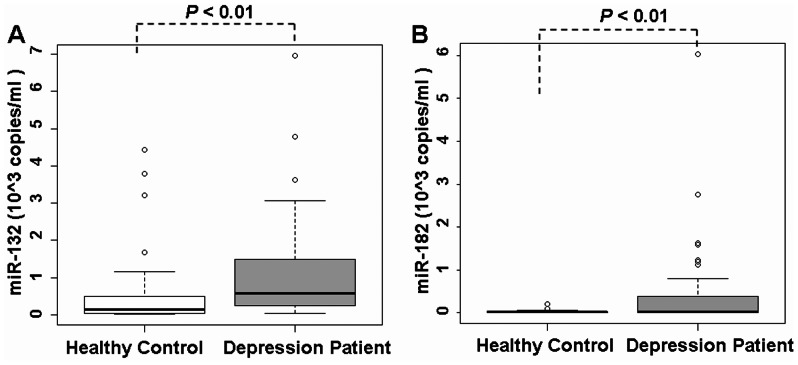
Serum miR-132 and miR-182 levels detected by real-time PCR. (**A, B**) Serum miR-132 or miR-182 levels in patients with depression and their controls, respectively. Real-time PCR showed that serum miR-132 (or miR-182) levels in depressed patients (n = 40) were much higher than those in healthy controls (n = 40, *P*<0.01).

Furthermore, we investigated the serum levels of the novel putative BNDF-regulating miR-182, along with miR-132, in our characterized human subjects by real-time PCR. We found that the serum miR-132 levels in patients with depression were 0.58×10^3^ copies/ml (n = 40), which were significantly higher than those in the serum of healthy controls (0.13×10^3^ copies/ml, *P*<0.01, n = 40, Table 1, Fig. 3A). In addition, the serum miR-182 levels in patients with depression were 0.02×10^3^ copies/ml (n = 40), which were also significantly higher than those in the serum levels of healthy controls (*P*<0.01, Table 1, n = 40, Fig. 3B). Our results demonstrated that miR-182 was a novel putative BNDF-regulating miRNA, which may be involved in the pathogenesis of depression.

### Relationship between the Serum Levels of BDNF and its Regulatory miRNAs

**Figure 4:**
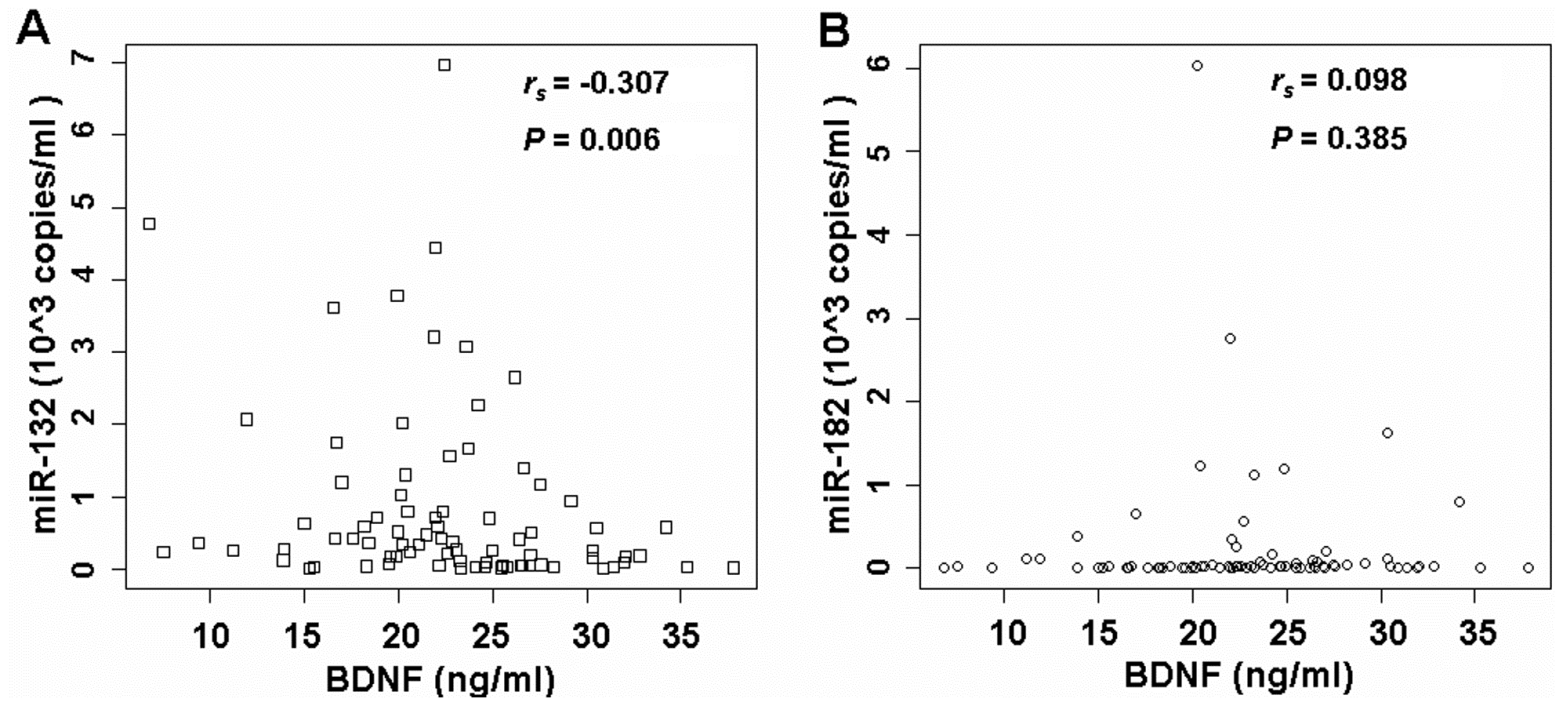
Relationship between levels of serum BDNF and its related miRNAs. (**A**) A significant negative correlation (Spearman *r_s_* = −0.307, *P = *0.006) between the serum BDNF and miR-132 levels in depressed patients (n = 40) and controls (n = 40). (**B**) No significant correlation (Spearman *r_s_* = 0.098, *P = *0.385) was found between the serum BDNF and miR-182 levels in depressed patients (n = 40) and controls (n = 40).

We have identified that the serum BDNF levels were decreased and the BDNF-related miRNAs including miR-182 and miR-132 were increased in human subjects with depression compared with control subjects. Due to the reported role of miRNAs in negatively regulating their targeting gene expression, we reasoned that there might exist a direct negative relationship between the serum BDNF levels and its-related miRNAs. To test this hypothesis, the relationship between the serum levels of BDNF and its-related miRNAs was examined by calculating Spearman’s correlation coefficient. Our results showed a significant negative correlation (Spearman *r_s_* = −0.307, *P = *0.006) between the serum BDNF levels and the miR-132 levels in patients with depression (n = 40) and controls (n = 40, Fig. 4A). However, there was no significant correlation (Spearman *r_s_* = 0.098, *P = *0.385) between the serum BDNF levels and the miR-182 levels in all patients (n = 40) and controls (n = 40, Fig. 4B).

### Relationship between SDS and the Serum Levels of BDNF-related miRNAs

**Figure 5:**
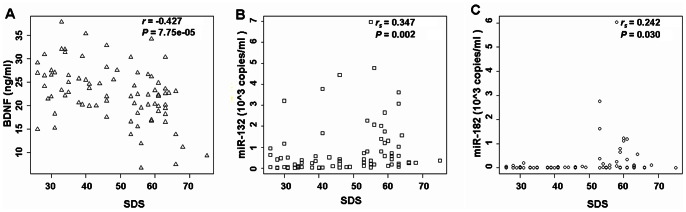
Relationship between serum levels of BDNF-related miRNAs and SDS score. (**A**) A significant negative correlation (Pearson *r* = −0.427, *P = *7.75E-05) was found between serum BDNF levels and SDS score in depressed patients (n = 40) and controls (n = 40). (**B**) An obvious positive correlation (Spearman *r_s = _*0.347, *P = *0.002) was revealed between the serum miR-132 levels and SDS score in patients with depression (n = 40) and controls (N = 40). (**C**) There was an obvious positive correlation (Spearman *r_s = _*0.242, *P = *0.030) between the serum miR-182 levels and SDS score in depressed patients (n = 40) and healthy controls (N = 40).

Finally, we studied whether the serum levels of miRNAs may be applied in diagnosing depression. The relationship between the serum levels of miRNAs and the Self-Rating Depression Scale (SDS) score was investigated by calculating Pearson’s (or Spearman’s) correlation coefficient. A significant negative correlation was revealed (Pearson *r* = −0.427, *P = *7.75E-05) between the serum BDNF levels and the SDS scores in all subjects (n = 80), including 40 patients with depression and 40 controls (Fig. 5A). In addition, there was a significant positive correlation (Spearman *r_s = _*0.347, *P = *0.002) between the serum miR-132 levels and the SDS scores in all subjects (n = 80), including 40 patients with depression and 40 controls (Fig. 5B). A significant positive correlation (Spearman *r_s = _*0.242, *P = *0.030) was also found between the serum miR-182 levels and the SDS scores in all subjects (n = 80, Fig. 5C). Thus, our results indicated that the serum levels of miRNAs were statistically correlated with the SDS scores, and suggested that their levels should be able to assist in the diagnosis of depression.

## Discussion

In current study, we characterized the roles of BDNF and its related miRNAs in the pathogenesis of depression. BDNF is an important member of the neurotrophin family of growth factors, which induces stress in both animal models [28] and human psychopathologies, including major depression, or posttraumatic stress disorder [10], [29]. We identified miR-132 and miR-182 as BDNF regulatory miRNAs by bioinformatics, and functionally validated their role in the negative regulation of the BDNF expression using a human neuronal cell model. Then we further explored the roles of BDNF and its related miRNAs in patients with depression and control subjects. Our results showed that the serum BDNF levels in patients with depression were significantly decreased compared with controls. Interestingly, the serum levels of miR-132 and miR-182 were increased in patients with depression, compared with their controls. A negative relationship between BDNF and miR-132 was found in serum of both patients and controls. There was also a negative correlation between the BDNF levels and the SDS scores in depressed patients and healthy controls. Moreover, significant positive correlations were found not only between the serum miR-132 level and SDS score, but also between the serum miR-182 levels and the SDS scores in depressed patients and healthy controls.

Identifying biomarkers and applying them in the diagnosis of major depressive disorder have remained a goal of clinicians and scientists for many decades. Karege F. and colleagues [30] demonstrated that cortical BDNF levels were consistent with serum BDNF levels in rodents and they also showed that serum BDNF levels were lower in patients with depression than those in matched controls [16]. Other reports have also indicated that serum BDNF levels were lower in drug-free major depressed subjects than in control subjects [31], [32]. Similarly, we found that serum BDNF levels were lower in patients with depression compared with controls. We also found that a negative correlation between BDNF level and SDS score in both patients and controls. Thus, the above studies suggest that serum BDNF may be a clinically useful biomarker for depression.

Despite the consistence from many findings showing lower serum BDNF levels in patients with depression, decreased BDNF levels have also been found in several other disease, like Huntington’s disease [33], Alzheimer’s disease [34], schizophrenia [35], and bipolar disorder [36]. Thus, the apparent lack of diagnostic specificity in the BDNF levels is likely to be a major drawback, which limits the true clinical application of this measure. Therefore, serum BDNF-related miRNAs, combined with serum BDNF levels, were investigated in this study to increase the feasibility of applying BDNF-related miRNAs in the diagnosis of depression. Our study also warrant future studies of the levels of these BDNF-related miRNA in other mental disorders.

A large number of miRNAs are shown to be specifically expressed in neuronal and non-neuronal cells in the brain and enriched in the central nervous system, and their expressions are regulated at various biological circumstances [24]. miR-124 and miR-128 are primarily expressed in neurons, whereas miR-23, miR-26, and miR-29 are expressed in high amounts in astrocytes [37]. During acute or chronic stress, the expression of miR-134, miR-183, miR-132, Let-7a-1, miR-9-1, and miR-124a-1 is altered in a hippocampal region responsible for mood [38]. miR-124 and cAMP response element-binding protein (CREB) are found to be involved in a feedback loop contributing to neuronal plasticity [39], which shows that miR-124 can control serotonin-induced synaptic facilitation through posttranscriptional suppression of CREB and, conversely, CREB can further regulate the expression of miR-124 [40]. Likewise, a similar mechanism has been identified between BDNF and miR-132, BDNF can dramatically up-regulate neuronal expression of miR-132 [41], while suppression of miR-132 by 2′-O-methyl oligoribonucleotide can increase BDNF transcript levels [27]. Similarly, our results demonstrated that miR-132 treatment in human neuronal cells significantly decreased the BDNF expression. Thus, these studies indicate that there may be a feedback loop between the expression of miR-132 and BDNF.

Our study further studied the neurobiological role of miR-132. MiR-132 is important for enhancing neuronal survival and its expression is up-regulated in transplanted neurons [42]. miR-132 expression in the murine prefrontal cortex can regulate neuro-developmental processes, but its dysregulation contributes to the neuro-developmental pathologies in schizophrenia [43]. Davis *et al*. reported that spontaneous cortical levels of miR-132 were lower at the end of the sleep-dominant light period than those at the end of the dark period in rats, suggesting that miR-132 played a regulatory role in sleep [44]. In this study, our results showed that the serum miR-132 levels were increased in patients with depression than those in controls, which might contribute to the lower serum BDNF levels.

We also investigated the biological roles of miR-182 in depression. miR-182 has been shown to regulate cell growth and suggested as a tumor-suppressive gene. Dysregulation of miR-182 can result in tumorigenesis, and lead to gastric adenocarcinoma through a mechanism targeted by CREB1 [45]. Over-expression of miR-182 expression can regulate cell cycle and suppress proliferation of lung cancer cells *in vitro* [46]. However, Bai *et al*. reported different roles of miR-182 in non-sonic hedgehog medulloblastoma, and showed that over-expression of miR-182 contributed to leptomeningeal metastatic dissemination in non-sonic hedgehog medulloblastoma and the knockdown of miR-182 decreased cell migration *in vitro* [47]. miR-182 has also been reported to involve in circadian rhythms in major depression patients with insomnia [22]. In this study, our results identified and characterized that miR-182 was a novel putative BDNF regulatory miRNA in a human neuronal cell model by both bioinformatic and molecular biological strategies. We also found that the serum levels of miR-182 were higher in patients with depression compared with those in healthy controls. The serum levels of miRNAs (miR-132 and miR-182) were found to be increased and BDNF levels were reduced in depressed patients compared with healthy controls, which supported that miR-132 and miR-182 could negatively regulate BDNF expression. Therefore, we found that a negative correlation between the serum BDNF levels and the miR-132 levels in depressed patients and healthy controls, but we did not found a significant negative correlation between the serum BDNF levels and the miR-182 levels in patients and controls. The relatively small number of available studies may lead to this limitation.

In summary, we characterized the roles of BDNF and its related miRNAs in the pathogenesis of depression and validated that miR-182 was a novel miRNA that regulated the BDNF levels. Our results also demonstrated that the serum BDNF levels were decreased, while the levels of miR-132 and miR-182 were increased in depressed patients compared with healthy controls. Our results clearly indicate that BDNF and its related miRNAs play important roles in the pathogenesis of depression. Further studies in larger and different subjects are warranted for more conclusive results.

## Materials and Methods

### Subjects

This study was performed at the outpatient department of Psychiatry of Yantai Fushan District People’s Hospital in China. The research protocol was approved by the Medical Ethics Committee of Binzhou Medical University. All experiments were performed in accordance with relevant guidelines and regulations of the Medical Ethics Committee of Binzhou Medical University. Prior to inclusion, eligible patients with depression, or their legal relatives in case of incapacity, provided written informed consent after study procedures were fully explained.

Chinese classification of mental disorders (CCMD-3) was performed for diagnosis, which was based on the Clinical descriptions and diagnostic guidelines of ICD-10. The diagnostic criteria also refer to the Research Criteria of ICD-10, and the DSM-IV [48]. Forty patients with depression, aged 23–61 years old, fulfilled the CCMD-3 criteria for depressive disorder and were diagnosed with depression first time. They did not take anti-depression drugs before. A careful diagnostic assessment was performed to rule out schizophrenia, paranoid psychosis, schizoaffective disorder, bipolar disorder, organic brain syndrome, chronic alcohol or drug abuse, and clinically relevant somatic illness. Then, they were evaluated by Self-Rating Depression Scale (SDS). SDS is a 20-item self-reported measurement of the symptoms of depression, which including statements about cognitive, psychomotor, somatic, and affective symptoms. Each item is scored from 1 to 4. The raw score is converted into standardized score. A cut-off higher than 53 is defined presence of depression according to the Chinese version of this scale (Zhang M, Handbook of psychiatry scales. Science and Technology Publishing Company of Hunan). Forty healthy controls, who came to Yantai Fushan District People’s Hospital for physical examination during the period from March 1st, 2010 to September 30th, 2010, were diagnosed without any mental or physical illness. All the controls provided written informed consent and agreed to participate in this study after study procedures were fully explained.

### Serum BDNF Detection

Serum samples from the patients with depression and controls were collected between 8∶00 and 9∶00 a.m. After centrifugation for 20 min at 2,650 g, serum was stored at 80°C. Serum BDNF was analyzed in duplicate as follows: The samples were incubated in a 96-well plate, followed by the addition of rabbit anti-human antibodies against BDNF (SC-546; Santa Cruz Biotechnology, Santa Cruz, CA) according to the manufacturer's instructions. Then antibody binding was detected by incubation with goat anti-rabbit IgG/HRP-labeled secondary antibodies (1∶2000, Beijing Zhong Shan-Golden Bridge Technology Co., Ltd., China) and their substrates in turn. The optical density of the reaction was estimated spectrophotometrically at 450 nm using an ELX800 ELISA reader (Bio-Tek Instrument Inc., USA).

### Real-time PCR

The mirVana™ miRNA isolation kit (Ambion, USA) was used to extract miRNAs from plasma according to the manufacturer's instructions. Then, miRNAs were added ploy (A) using poly (A) polymerase (Ambion). The cDNAs were synthesized by RT primer 5′-AACATGTACAGTCCATGGATGd(T)30N(A,G,C or T)-3′, miR-132 and miR-182 were analyzed by real-time PCR. The miR-132 forward primer: 5′-ACAGTCTACAGCCATGGTC-3′. Reverse primer was 5′-AACATGTACAGTCCATGGATG-3′. The miR-182 forward primer was 5′-GGCAATGGTAGAACTCACACT-3′. Reverse primer was 5′-AACATGTACAGTCCATGGATG -3′. Real-time PCR analysis was performed by SuperTaq Polymerase (Takara, Japan). The expression of miRNAs was detected using the RG3000 system (Corbett Research, Austrilia) with the Quantitect SYBRGreen Kit (Qiagen) as our previous study [49], [50]: an initial denaturation at 95°C for 4 min, followed by 40 cycles of 95°C denaturation for 30 s, 52°C annealing for 30 s, and extension at 72°C for 30 s. Fluorescence was detected at 585 nm at each extension step of 72°C. Human 5S rRNA was added into each sample and served as control. All transfections were performed in triplicate.

### Cell Culture and Transfection

Human neuroblastoma cells (SH-SY5Y, obtained from Shanghai Institute of Cell Biology, China) were grown in DMEM/F12 medium (Hyclone, USA) containing 10% FCS (Hyclone, USA) and 100 U/ml of penicillin-streptomycin (Sigma, USA) at 37°C with 5% CO2.

MiR-132 and miR-182 were chemically synthesized in the form of small interfering RNA (siRNA) duplexes according to Park’s study [51] (Table 2). 1×10^6^ cells were transfected with 0.5 µg miRNA in 1.5 µl of lipofectamine 2000 (Invitrogen, USA), according to the manufacturer’s instructions. All transfections were performed in triplicate.

**Table 2:** The sequences of chemically synthesized miRNA^a^.

Oligos		Sequences (5′→3′)
miR-132	sense	uaacagucuacagccauggucg
	antisense	cgaccauggcuguagacuguuauu
miR-182	sense	uuuggcaaugguagaacucacacu
	antisense	agugugaguucuaccauugccaaauu
control (scramble sequence)	sense	caguacuuuuguguaguacaa
	antisense	guacuacacaaaaguacuguu

a The selected miRNAs were chemically synthesized in the form of small interfering RNA (siRNA) duplexes.

### Western Blotting

Forty-eight hours after transfection, total protein lysates of SH-SY5Y cells were prepared and each 40 µg protein sample was loaded onto 10% SDS-PAGE. After electrophoresis, the protein was transferred to PVDF membrane, which was blocked with 7% non-fat milk in TBST [50 mmol/L Tris-HCl (pH 7.6), 150 mmol/L NaCl, 0.1% Tween-20] for 1.5 h at room temperature. The TBST buffer solution was added to wash the membrane 3 times before immunoblotting with polyclonal rabbit anti-human BDNF antibody (1∶400, SC-546; Santa Cruz Biotechnology, Santa Cruz, CA). After being incubation at 4°C overnight and a wash with TBST, the membrane was incubated with a HRP-labeled goat anti-rabbit IgG (1∶4000, Beijing Zhong Shan-Golden Bridge Technology Co., Ltd, China) for 1 h at room temperature. The same membrane also was stripped and re-probed with Actin antibody (1∶400, Beijing Zhong Shan-Golden Bridge Technology Co., Ltd, China) as control. All experiments were performed in triplicate.

### Statistics

Data were first tested for normal distribution and variance homogeneity with shapiro.test and F test, respectively. If normal distribution was verified, data were expressed as means ± SD, or expressed as median and quartiles. Since BDNF and SDS score showed normal distribution, differences between these two groups were analyzed using Student's t-test and Pearson's correlation coefficient was calculated to assess the relationship among variables. Since miR-132, miR-182 did not showed normal distribution, nonparametric tests were applied. Differences in continuous variables (miR-132, miR-182) between groups were analyzed by Wilcoxon rank sum test, and the correlations were calculated by the Spearman rank test. Statistical analyses were performed with R version 2.15.0 (Copyright (C) 2012, ISBN 3-900051-07-0). For all statistical analyses, *P*<0.05 was considered statistically significant.
